# Pathological Characterization of African Swine Fever Viruses With Genetic Deletions Detected in South Korea

**DOI:** 10.1155/tbed/9917280

**Published:** 2025-05-06

**Authors:** Seong-Keun Hong, Ki-Hyun Cho, Jung-Hoon Kwon, Da-Won Kim, Jongho Kim, Da-Young Kim, Hae-Eun Kang, Jong-Soo Lee, Yeon-Hee Kim

**Affiliations:** ^1^Foreign Animal Disease Division, Animal and Plant Quarantine Agency, 177 Hyeoksin 8-ro, Gimcheon 39660, Republic of Korea; ^2^College of Veterinary Medicine, Chungnam National University, 99 Daehak-ro, Yuseong-gu, Daejeon 34134, Republic of Korea; ^3^College of Veterinary Medicine, Kyungpook National University, 80 Daehak-ro, Buk-gu, Daegu 41566, Republic of Korea; ^4^Animal Disease Diagnostic Division, Animal and Plant Quarantine Agency, 177 Hyeoksin 8-ro, Gimcheon 39660, Republic of Korea

**Keywords:** African swine fever, animal experiment, genetic characterization, next-generation sequencing, pig farms, South Korea, virulence

## Abstract

African swine fever virus (ASFV) genotype II has been circulating in South Korea, causing substantial economic losses to the Korean pig industry since 2019. Genetic epidemiological investigations using whole-genome sequencing have been conducted to track the genetic evolution of ASFV. Two ASFV strains were detected in domestic pig farms in South Korea, one with a large deletion in the MGF 360-6L gene and the other in the MGF 360-21R gene. Phylogenetic analysis indicated that all Korean isolates belonged to the Asian subgroup of ASFV genotype II and were further divided into distinct subclusters of Korean African swine fever (ASF) group I. To identify the pathological changes caused by the deletion of MGF 360-6L and MGF 360-21R genes, we evaluated their pathogenicity in experimentally infected domestic pigs. No significant changes in pathogenicity were observed compared to other viruses evaluated in our previous studies. All inoculated pigs died 7–10 days post-inoculation (dpi), showing acute forms of illness with common pathological lesions. These results highlight that large genetic deletions can occur naturally in ASFV, but the deletions in MGF 360-6L and MGF 360-21R genes did not alter pathogenicity in domestic pigs. Further research is needed to understand the roles of these genes, especially in viral replication and pathogenicity in wild boars and ticks.

## 1. Introduction

African swine fever (ASF) is a disease caused by a highly contagious hemorrhagic virus that spreads rapidly among both domestic and wild boars, regardless of their age [1, 2]. This devastating illness has significant implications for the economic and productive sectors, resulting in substantial losses of revenue and output. ASF was first reported in Kenya in 1921 [3]. Recently, ASF was prevalent in countries in sub-Saharan Africa and the region of Sardinia in Italy. Since its inception in Georgia in 2007, ASF has spread to Russia and Eastern Europe. In 2018, China reported its first ASF outbreak [4], and the disease has since spread to neighboring Asian countries, including Mongolia, Vietnam, Cambodia, North Korea, Myanmar, Laos, the Philippines, Indonesia, Timor-Leste, Papua New Guinea, India, Bhutan, and Thailand (WOAH–WAHIS interface).

The disease etiology is attributed to the ASF virus (ASFV), a large double-stranded DNA virus belonging to the Asfarviridae family. ASFV exhibits a wide range of clinical manifestations and is associated with high mortality in domestic pigs, with acute forms characterized by high fever (up to 40°C), depression, and hemorrhagic manifestations. ASFV genotyping involves examining specific genetic markers, such as the major p72 encoded by the *B646L* gene and the central variable region (CVR) within *B602L*. ASFV can be subtyped using sequence information from the p54 (*E183L*), CD2v (*EP402R*), and CVR of *B602L* and tandem repeat sequence region between the *I73R* and *I329L* genes [5–7]. Analysis of other genes, such as *O174L*, *K145R*, and *MGF 505-5R*, the *CP204*L encoding p30 protein, Bt/Sj region, *J268L* region, and intergenic regions (IGRs) between the *A179L* and *A137R* and between the *MGF 505-9R* and -*10R* region can be used to differentiate between closely related ASFV strains [8]. ASFV strains have been categorized into 24 genotypes [5, 9, 10], with genotypes I and II being identified outside Africa.

A recent reevaluation of six distinct p72 groups has been proposed. It highlights that most p72-based genotypes were initially formulated without being classified under any specific methodological framework or accurately compared to preexisting ASFV genotypes [11].

Genotype II ASFV strains were first reported in Georgia in 2007 [12] and subsequently spread to Europe and the Caribbean, causing a global pandemic in 2014 [13]. However, in Asia, China reported the presence of genotype I ASFV isolated for the first time in 2021 and a genotype II ASF outbreak on a pig farm in 2018, followed by outbreaks in numerous Asian countries [4, 14, 15].

In South Korea, genotype II ASF was first reported in 2019 on a pig farm in Paju, Gyeonggi Province [16]. A total of 38 ASF cases were reported in domestic pig farms, predominantly in the northern regions, between September 16, 2019 and September 25, 2023. On October 3, 2019, an ASFV-infected wild boar was discovered in the demilitarized zone of the northwestern border region. Despite concentrated governmental efforts to control the spread of wild boars, the regions inhabited by infected wild boars have continued to expand towards the east and south along the mountainous terrain [17, 18]. As of December 31, 2023, a cumulative total of 3486 infected wild boars have been officially detected across Gyeonggi, Gangwon, Chungcheongbuk, and Gyeongsangbuk provinces.

In a previous study, 12 gene markers were examined using partial sequencing [19]. Recently, whole-genome sequencing using advanced sequencing techniques, next-generation sequencing (NGS), has been used for comprehensive genomic sequencing of various viruses, including ASFV [20–24]. Whole-genome sequencing of ASFVs enables the detection of all genetic variations and can be used to analyze viral evolution and genomic epidemiology [25].

In South Korea, ASF surveillance strategies focus on the prompt identification of acute cases, with an emphasis on clinical surveillance that facilitates early detection and diagnosis, primarily through antigen detection [26]. Any significant changes in the virulence of circulating ASFV isolates would necessitate adjustments to the existing control strategies. To address this, the Animal and Plant Quarantine Agency in South Korea conducted NGS analysis to detect genetic polymorphisms among viruses and monitor pathogenicity changes in domestic pigs. In this study, we detected two ASFVs from domestic pig farms in Pocheon and Cheorwon provinces that exhibited unusual genetic mutations: one with a large deletion in the MGF 360-6L gene and another in the MGF 360-21R gene. We analyzed the whole genome of the virus and evaluated the pathological changes by examining clinical signs, disease progression, and postmortem lesions observed in ASF-infected pigs.

## 2. Materials and Methods

### 2.1. Viruses

Two ASFVs with unusual genetic mutations were detected from domestic pig farms in the Pocheon and Cheorwon provinces. The two isolates were designated ASFV/Korea/Pig/Pocheon2/2023 and ASFV/Korea/Pig/Cheorwon2/2023 (Table 1). The Pocheon strain reported on March 19, 2023, was detected from an ASFV-infected pig farm following a farmer's notification and was isolated from the spleens of dead pigs. The Cheorwon strain, reported on July 18, 2023, was detected through active surveillance of antemortem inspection of pigs and isolated from the blood of infected pigs. No clinical manifestations were observed, but diagnosis revealed that three of 21 ASFV-positive pigs had 22–29 cycle threshold (Ct) value by WOAH TaqMan quantitative polymerase chain reaction (qPCR) [27]. The virus was isolated from the samples according to the procedure of the Center for Animal Health Research, European Union Reference Laboratory of ASF, and as previously described [28].

### 2.2. DNA Extraction and Real-Time PCR Analysis

Automatic DNA extraction was carried out in accordance with the Maxwell RSC 48 instrument using Maxwell RSC whole blood DNA and Maxwell RSC viral total nucleic acid purification kit (Promega, Madison, WI, USA), following the manufacturer's instructions. Viral genome load was estimated by correlating with Ct values using qPCR. WOAH TaqMan qPCR targeting the *B646L* gene encoding p72 was performed using Bio-Rad CFX-96 (Bio-Rad Laboratories, Hercules, CA, USA) as described in the WOAH Manual [29]. Samples with Ct < 40.0 were considered positive, while those without Ct values were considered negative.

### 2.3. Whole-Genome Sequencing and Data Analysis

The isolated viral genomes were analyzed by NGS after ASFV genome target enrichment. ASFV gene enrichment was performed by hybridization with capture probes using the Celemics Target Enrichment Kit (Celemics, Seoul, Korea). DNA sequencing libraries for the Illumina MiniSeq (Illumina, San Diego, CA, USA) were prepared following the manufacturer's instructions using Nextra XT DNA Library Preparation Kit and the Netra XT Index Kit v2 Set A (Illumina). Library quality was assessd using a BioAnalyzer 2100 (Agilent, Santa Clara, CA, USA) with an Agilent Bioanalyzer DNA High Sensitivity kit (Agilent). Quantification was performed using a double-stranded DNA High Sensitivity Assay kit (Thermo Fisher Scientific) and a Qubit 2.0 Fluorometer (Thermo Fisher Scientific). MiniSeq sequencing in 150 bp paired-end mode was conducted using the MiniSeq High Output Reagent kit (300-cycle; Illumina) following the manufacturer's instructions.

Adapter sequences and low-quality sequencing reads with a quality score <70 were removed using the BBDuk v38.84 program. Taxonomy classification was done using the KRAKEN2 program (https://ccb.jhu.edu/software/kraken2/) to evaluate the ASFV genome proportion in the sample. The trimmed sequencing reads were mapped against the ASFV Georgia 2007/1 gene (GenBank Accession number NC_044959) using Geneious Prime software (https://www.geneious.com), allowing up to 10% mismatch. Consensus sequences were generated based on sites with coverage depths of >20. The NGS and assembly data are presented in Table S3. The assembled whole genome of the virus was submitted to GenBank database with accession numbers PQ185530 and PQ185531.

### 2.4. Phylogenetic Analysis

Whole genome sequences of genotype II ASFVs available in GenBank (https://www.ncbi.nlm.nih.gov/genbank/; data assessed on 13 August, 2024) were downloaded. Sequences exhibiting an unusually high number of mutations, exceeding the typical rate of viral mutations, were excluded due to the potential for sequencing errors. For efficient computation and visualization, we selected representative sequences from those redundantly reported in the same region and during similar periods. Furthermore, we reduced the number of European ASFV sequences for clearer visualization based on previous studies [25], indicating that European viruses are phylogenetically distinct from the Korean isolates. A total of 119 reference genomes, including Georgia 2007/1 strain, were selected for phylogenetic analysis (Table S2). The two ASFV genomes detected in this study were aligned with the reference genomes by multiple alignments using fast Fourier transform method and manually trimmed to equal lengths using Georgia 2007/1. G/C homopolymers and inverted terminal repeats, prone to sequencing errors, were excluded. Part of the genome exhibiting large size gene deletions or replacements, detected in the isolated virus, was also excluded from the phylogenetic analysis. A maximum likelihood phylogenetic tree was constructed using RaxML v8.2.7 by employing a general time reversible (GTR) nucleotide substitution model. Bootstrap analysis with 500 replicates assessed the statistical support of the phylogenetic tree, with Georgia 2007/1 as the root. Furthermore, a time-scaled phylogenetic tree was constructed using the BEAST v1.10.4 [30], employing an uncorrelated relaxed clock model with gamma-distributed rate (GTR + *γ*) nucleotide substitution. Four Markov chain Monte Carlo runs, each comprising 100 million steps, were performed in parallel. The parameters and trees were sampled every 10,000 steps, resulting in 10,000 parameter states and posterior trees. TRACER v1.7.2 [31] was used to analyze the parameters, with 10% of each result discarded as burn-in. All parameters had an effective sample size >200. A time-scaled maximum clade credibility tree was generated using TreeAnnotator v1.10.4 (https://beast.community/treeannotator) in BEAST and visualized using FigTree v1.4.4 (http://tree.bio.ed.ac.uk/software/figtree/).

### 2.5. Experimental Design in Animals

For the in vivo studies, 13 8-week-old domestic pigs of the Landrace strain from a commercial pig farm, both sexes, were infected with different ASFV strains in two independent trials. Commercial pig farm is a farrow-to-finish farm which was free of ASF and specific swine pathogens. The disease status and vaccine program were as previously described [28]. The experiments were performed in the Biosafety Facility (Animal Biosafety Level 3; ABSL3) of the Animal and Plant Quarantine Agency (APQA), Gimcheon, South Korea. All experiments were performed in compliance with the protocols approved by the Institutional Animal Care and Use Committee of APQA (authorization no. 2023-815, approved on December 11, 2023) to ensure ethical and humane treatment of the animals. Before infection, the health status of all animals was evaluated and confirmed to be free of ASFV through veterinary examination. Ten pigs were inoculated intramuscularly with ASFV at a titer of 10^3^ HAD_50_/mL. The remaining three pigs were treated as the mock-infected group. During the experiment, the room temperature was maintained at 23.0 ± 2.0°C with 50.0 ± 20.0% humidity. All procedures, including euthanasia, were performed following the endpoint criteria of the actual legal regulations and dead pigs were immediately subjected to necropsy.

### 2.6. Clinical Assessment and Sample Collection

Clinical signs and rectal temperatures were recorded daily from the day of viral inoculation until death. The clinical signs in each pig were scored according to previously described guidelines with minor modifications [32]. All samples were collected on a daily basis; whole blood sampling was performed via a syringe at the neck, which was collected by K_2_EDTA tubes (18.0 mg EDTA, BD Vacutainer, Plymouth, UK). Oral, nasal, and rectal swabs were collected in Clinical Virus Transport Medium (CTM Plus; Noble Bio, Hwaseong, Republic of Korea) using sterile cotton swabs. All pigs were subjected to necropsy immediately following death or euthanasia and 12 tissue samples (liver, spleen, submandibular/tracheobronchial/gastric/gastrohepatic/renal/mesenteric lymph nodes, kidney, heart, thoracic vertebrae, and hindlimb muscles) were collected to determine the number of ASFV genome copies. All samples were analyzed to assess ASFV load using qPCR.

### 2.7. Histopathology

During necropsy, samples from the tonsils, spleen, liver, kidneys, lungs, small and large intestines, urinary bladders, thoracic spinal cords, and lymph nodes (submandibular, tracheobronchial, gastric, gastrohepatic, renal, and mesenteric) were collected for histological analysis. The samples were fixed in 10% neutral-buffered formalin and processed routinely by hematoxylin and eosin (H&E) staining. All histological changes were classified into four categories: normal (0), mild (1), moderate (2), and severe (3), according to ASF pathology guidelines [32].

### 2.8. Statistical Analysis

Data are presented as means ± standard deviation (SD) from at least five independent experiments (*n* = 5). Statistical differences between the groups were determined by performing an analysis of variance (ANOVA) using GraphPad Prism 8 software (GraphPad Software, Inc, San Diego, CA, USA) and Tukey's multiple comparisons test to compare groups. The means of the single groups were compared using the Student's *t*-test. Statistical significance was denoted as *p* value of *⁣*^*∗*^*p* < 0.05 and *⁣*^*∗∗*^*p* < 0.01 for significant and highly significant, respectively.

## 3. Results

### 3.1. Whole Genome Comparative Analysis and Phylogenetic Analysis

**Figure 1:**
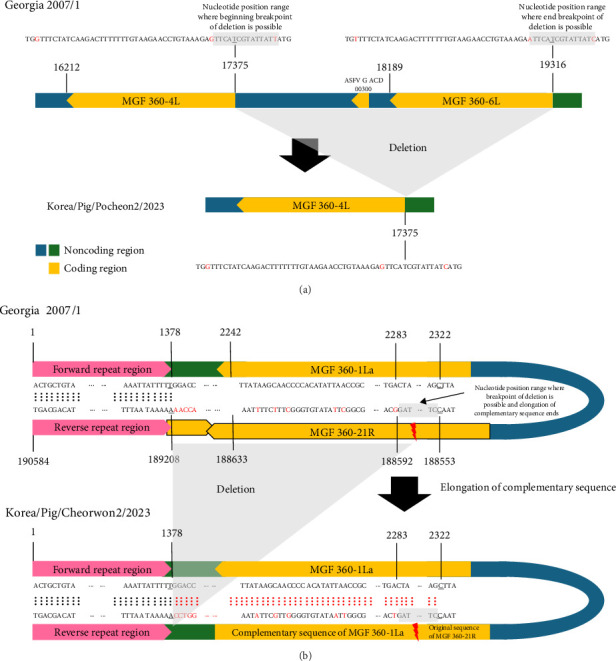
Diagram illustrating large gene deletion or replacement in the two ASFV isolates. (A) Deletion were identified within the 1942 bp segment with the nucleotides from end of MGF 360-4L gene to end of MGF 360-6L genes in the Pocheon strain. (B) Nucleotide position range where breakpoint of deletion is confirmed by cvPCR and elongation of complementary sequence ends in the Cheorwon strain.

To compare the two ASFV isolates from 2023, we performed a comparative analysis of their whole-genome sequences against the Georgia 2007/1 reference strain sequence (p72 genotype II). Large-size genetic deletions were detected in both isolates. In the ASFV/Korea/Pig/Pocheon2/2023 strain, 1941 bp deletion was observed from the 3′ end of the MGF 360-4L gene to the 3′ end of MGF 360-6L gene, resulting in the deletion of ASFV G ACD and MGF 360-6L CDS (Figure 1A). In the ASFV/Korea/Pig/Cheorwon2/2023 strain, the MGF 360-21R CDS was deleted and replaced with the complementary sequence of MGF 360-1 La (Figure 1B). These large size deletions and replacements were confirmed by Sanger sequencing (Table S1). Furthermore, two ASFV isolated showed 18 mutations, including single nucleotide polymorphisms (SNPs) and insertion/deletion polymorphisms (Indels). Detailed analysis denoted in Table 2.

**Figure 2:**
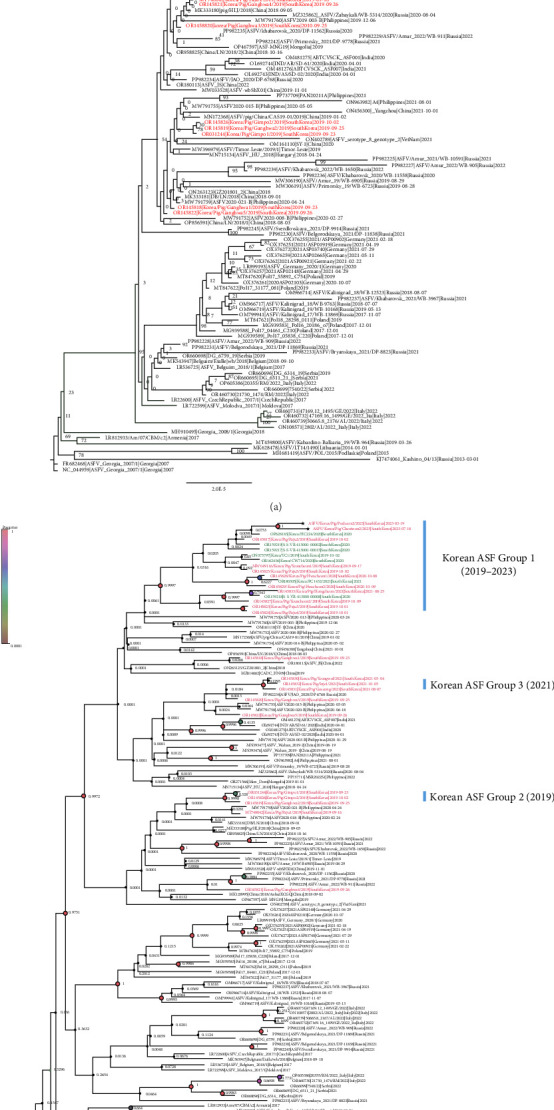
Phylogenetic trees of genotype II African swine fever viruses. Taxa with red font are the ASFV isolates from domestic pigs and green font are the wild boars in South Korea. Two 2023 isolates analyzed in this study are marked with star. (A) Maximum likelihood phylogenetic tree. Phylogenetic trees were constructed using the maximum likelihood method in RAxML with 500 bootstrap replicates. Bootstrap values are shown in each node. (B) Time-scaled maximum clade credibility tree. The size and the color of the node (circle) indicate the posterior, which means the probability associated with forming one clade. Korean ASF was divided into three groups according to the posterior probability.

A maximum likelihood phylogenetic tree (Figure 2A) and a time-scaled maximum clade credibility tree (Figure 2B) were constructed to examine the evolutionary relationships of the two novel ASFVs, both of which have large deletions. The maximum likelihood phylogenetic tree revealed that the two isolated viruses clustered with other Korean viruses isolated between 2019 and 2020 (Figure 2A). However, most nodes in the tree did not receive strong bootstrap support (<70%), indicating uncertainty in the branching patterns. To gain a more comprehensive understanding of the viral spread and evolution, we conducted a time-scaled Bayesian phylogenetic analysis.

A time-scaled phylogenetic tree revealed that the Korean ASFVs were divided into at least three subgroups (Korean ASF group I–III), with each subgroup sharing a common node supported by a high posterior probability (>0.9), consistent with the findings of a previous study [25] (Figure 2B). The 2023 isolates clustered with subgroup I viruses isolated between 2019 and 2020, suggesting that these viruses have evolved locally since their initial introduction in 2019.

### 3.2. Comparative Clinical Outcomes

**Figure 3:**
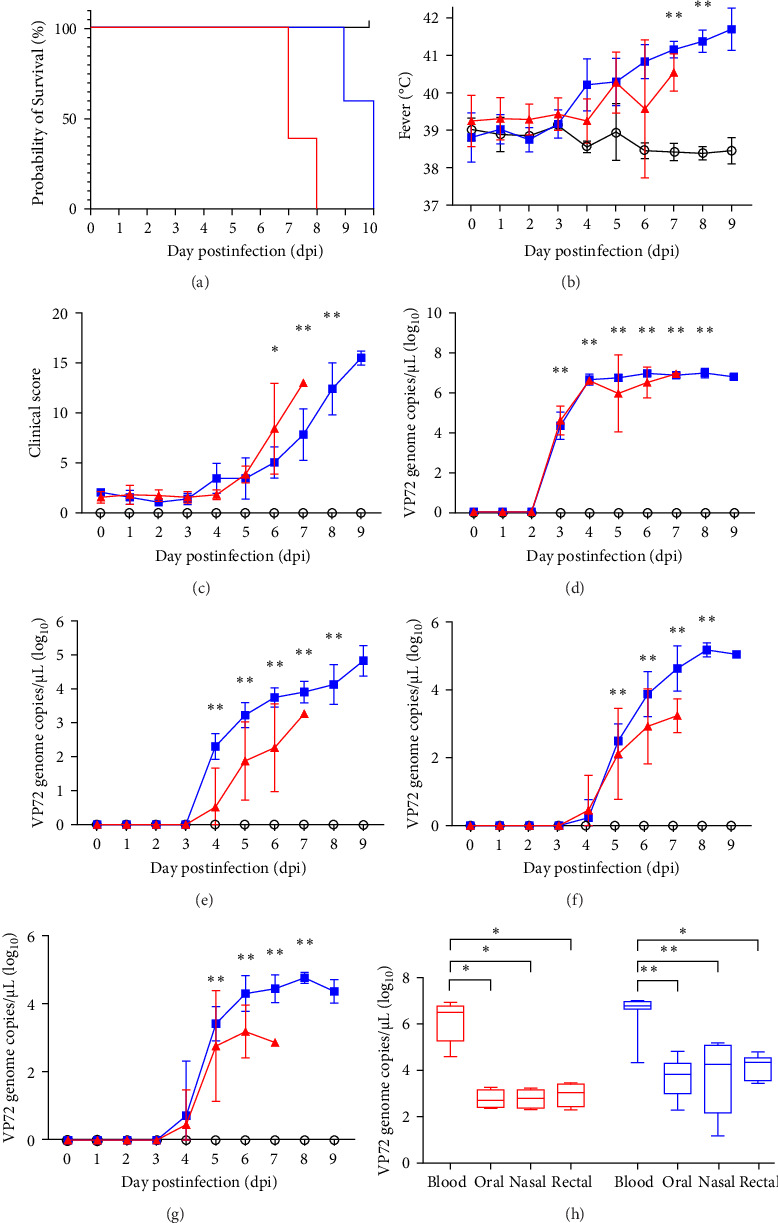
Comparative analysis of experimental pig groups in the two Korean isolates infection (red line; Pocheon2 strain (*n* = 5) and blue line; Cheorwon2 strain (*n* = 5)) and negative control groups (black line; *n* = 3). Results of (A) pig survival rates and the dynamics of (B) rectal temperature and (C) clinical score were denoted. Viremia and virus shedding condition were determined with viral copy number per 1 µL in (D) blood and experimental pig swab samples, including (E) oral, (F) nasal, and (G) rectal swabs. Also, (H) viral load compared to the swab samples versus blood. *⁣*^*∗*^*p*  < 0.05, *⁣*^*∗∗*^*p*  < 0.01.

All the inoculated animals were successfully infected independently and the survival rate and onset of death in the experimental pigs are shown in Figure 3. In mortality, Group 1 pigs died or were euthanized at 7.4 ± 0.5 days post-inoculation dpi and at 9.4 ± 0.5 dpi in Group 2 (Figure 3A). Inoculated pigs started to show high fever (>40°C) at 4.2 ± 1.8 dpi and 4.4 ± 0.9 dpi in Groups 1 and 2, respectively (Figure 3B). Comparative clinical scores of the two inoculated groups are shown in Figure 3C. Clinical signs were first observed at 6.4 ± 0.5 dpi and 6.6 ± 1.1 dpi in Groups 1 and 2, respectively, and the maximum clinical score immediately before death ranged from 11 to 16 points. All inoculated groups commonly displayed fever, loss of appetite, anorexia, depression, and recumbency. In Group 1, some pigs showed ocular discharge (2/5, 40%) and diarrhea (2/5, 40%), while Group 2 presented skin hemorrhage (2/5, 40%), joint swelling (1/5, 20%), wheezing/coughing (1/5, 20%), ocular discharge (5/5, 100%), and hematuria/epistaxia (1/5, 20%; Table 3). There were no significant differences in the mean total survival rate or clinical manifestations between the groups. As expected, the pigs in the mock group showed normal conditions and temperatures during the study period.

### 3.3. Comparative Analysis of Viral Genome Load in Blood and Virus Shedding

To investigate the time-dependent serial changes in viremia and excretion patterns of representative ASFV strains from the two experimental groups, blood, and virus shedding via the oral, nasal, and rectal routes were monitored until death. ASF viral genome was first detected in blood samples from Group 1 at 3.4 ± 0.9 dpi (mean 1.5 × 10^4^ copies/µL) and Group 2 at 3 dpi (mean 2.2 × 10^4^ copies/µL), which was 1–2 days earlier than the onset of pyrexia (expect pig 5 in Group 1). On the first day of detection in the blood, viral genome loads increased to more than 3.6 × 10^6^ copies/µL and 1 × 10^7^ copies/µL 1 day later. Notably, a high level of viral load was maintained in all the groups until the end of the experiment. Among all the experimental pigs, both groups were ASFV-positive as early as approximately 3 dpi. Furthermore, the viremia of the inoculated pigs in Groups 1 and 2 did not show significant differences in copy number.

In viral shedding, the average onset time of virus excretion from the oral route was 4.8 ± 0.5 dpi (6.9 × 10^1^ copies/µL) and 4.2 ± 0.4 dpi (3.3 × 10^2^ copies/µL), in Groups 1 and 2 respectively, in which, maximum titers of viral genome loads were 1.9 × 10^2^ copies/µL in Group 1 and 3.9 × 10^4^ copies/µL in Group 2. At the beginning, detection via the nasal and rectal route was observed at 5.0 ± 0.7 dpi (1.7 × 10^2^ copies/µL and 1.1 × 10^3^ copies/µL) and 4.8 ± 0.4 dpi (1.6 × 10^2^ copies/µL and 2 × 10^3^ copies/µL), respectively. The mean maximum titer was nasal swab at 1.7 × 10^5^ copies/µL in Group 2. Viremia and viral shedding via the three routes are shown as the average ± SD and the comparative results of the two groups are shown in Figure 3 and Table 4.

### 3.4. Comparative Analysis of Gross Pathology and Virus Presence in Tissues

To evaluate viral replication in tissues and gross pathological findings, we compared each group by necropsy and qPCR of dead pigs. All groups tested positive for the presence of ASFV in the subjected organs, including the tonsils, spleen, lymph nodes, heart, lungs, liver, intestine, kidneys, and urinary bladder (Table 5). The lymphoid system lesions commonly observed in all the pigs included hemorrhagic infarction in the spleen and splenomegaly. Also, the infected pigs mostly exhibited erythema (90%), enlargement (100%), and hemorrhagic infarction (80%) of the lymph nodes (submandibular, tracheobronchial, gastric, gastrohepatic, renal, and mesenteric). The cardiorespiratory system lesions included epi-/endocardial hemorrhage, petechiae (20%), and erythema (80%) of the heart. Three pigs showed interstitial pneumonia and four pigs showed interlobular edema. Furthermore, the alimentary and urinary systems varied, including petechiae of the liver and intestine in the serosa, hemorrhagic renal medulla, and petechiae on the renal cortex and mucosa of the urinary bladder. The types of genetic deletions in ASFVs were basically the same among all groups, expect for slight differences in the viral loads in tissues and pathological findings among individuals. The major gross macroscopic lesions in each pig are summarized in Table 6. The highest average viral genome loads were observed in the spleen (Group 1 at 2.7 × 10^6^ copies/µL and Group 2 at 1.3 × 10^6^ copies/µL), followed by the liver (Group 1 at 2 × 10^6^ copies/µL and Group 2 at 6.1 × 10^5^ copies/µL) and six lymph nodes (Group 1 at 4.5–5.7 × 10^5^ copies/µL and Group 2 at 2.6–4.4 × 10^5^ copies/µL), with no significant difference between each group (Figure S1).

### 3.5. Histopathological Analysis

**Figure 4:**
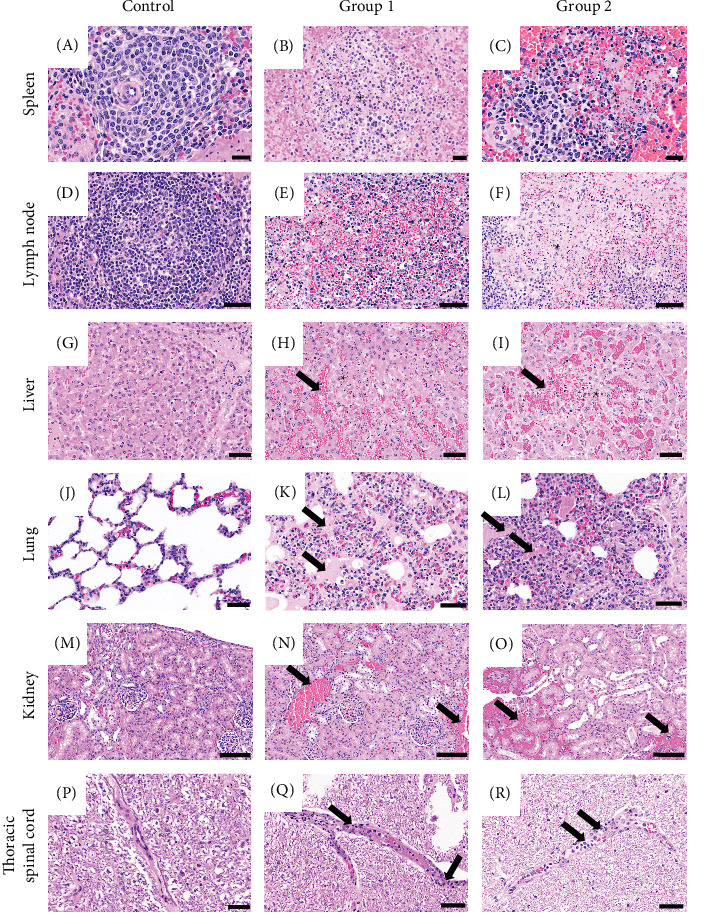
Histological examinations in pigs experimentally infected with two Korean isolates. (A–C) Spleen, scale bar = 20 µm; (A) normal white pulp and (B, C) severe lymphoid necrosis on asterisks. (D–F) Gastrohepatic lymph nodes, scale bar = 50 µm; (D) normal lymphoid follicle and (E, F) severe lymphoid necrosis with hemorrhages on asterisks. (G–I) Liver, scale bar = 50 µm; (G) normal hepatocytes and sinusoid and (H, I) focal necrosis of hepatocytes with hemorrhages on asterisks. (J–L) Lungs, scale bar = 50 µm; (J) normal alveolar spaces and walls and interstitial pneumoniae with edema (arrows) in (K) and (L). (M–O) Kidneys, scale bar = 100 µm; (M) normal glomerulus and interstitium and (N, O) renal hemorrhages (arrows). (P–R) Spinal cord, scale bar = 50 µm; (P) normal blood vessel in thoracic spinal cord and (Q, R) vascular degeneration with weak perivascular lymphoid cell infiltrations (arrows).

To identify whether the representative ASFV was affected in the tissues, we performed H&E staining of the tissues of infected pigs. The gross lesions observed in all infected pigs were splenomegaly with infarctions. Moderate to severe hemorrhagic enlargement in the lymph nodes (submandibular, gastrohepatic, gastric, and renal) were found in nine out of 10 pigs except for a pig (G1-5). Major gross lesions in ASFV-inoculated pigs are presented in Table 6 and Figure 4. The histological lesions in both groups of infected pigs were prominently moderate to severe necrotizing splenitis with hemorrhages. All pigs, except for one in Group 1 (Pocheon strain) had necrotizing lymphadenitis with hemorrhages from all lymph nodes, mild interstitial pneumonia with edema, and mild-to-moderate multifocal hemorrhages in the renal cortex and medulla. Furthermore, mild hepatocellular necrosis with periportal mononuclear cell infiltrations (Group 1, *n* = 3 and Group 2, *n* = 5), moderate lymphoid cell lysis in the tonsils (Group 1, *n* = 3 and Group 2, *n* = 3), and myocardial hemorrhages (Group 1, *n* = 1 and Group 2, *n* = 2) were observed. Notably, four pigs in each group displayed mild-to-moderate vascular degeneration with perivascular inflammatory cell infiltration. Consistent with gross findings, no histological differences were observed between the two groups.

## 4. Discussion

The ongoing presence of ASFV in annual outbreaks on pig farms and among wild boars in South Korea necessitates continuous monitoring and detection of any alterations in virulence. Identifying ASF in pigs may present challenges when the animals are exposed to a strain of ASFV that has not yet been thoroughly studied. However, determining whether adjustments to existing control strategies and surveillance protocols are needed is crucial. The ASFV strains found in South Korea belong to p72 genotype II, known for their high virulence in both domestic pigs and wild boars, causing severe disease with near-total lethality in animals of all ages and sexes [28, 33]. Furthermore, the virus can induce rapid mortality in infected hosts, leading to significant economic losses in the swine industry in Europe and Asia [34–36]. Therefore, ASFV is recognized as a major transboundary animal disease, reportable to WOAH, with frequent outbreaks worldwide, underscoring unresolved questions regarding its biology and immunology.

In this study, we identified the large-size deletions in both 2023 ASFV isolates, representing significant genomic changes. The ASFV/Korea/Pig/Pocheon2/2023 strain showed a 1941 bp deletion spanning from the 3′ end of the MGF 360-4L gene to the 3′ end of the MGF 360-6L gene. In the ASFV/Korea/Pig/Cheorwon2/2023 strain, we observed the potential for self-reversion in the MGF 360 gene, characterized by the deletion of the MGF 360-21R gene, which was replaced by the complementary sequence of MGF 360-1 La. Phylogenetic analysis indicated that these large genetic deletions in both viruses could have occurred during the circulation of the viruses in South Korea, as both strains clustered with previous Korean ASFV isolates. These large genetic deletions or reversions may occur due to template switching by the polymerase among DNA or RNA strands with high sequence identity [37]. The exact mechanism of large genetic deletions and reversions in viruses remains incompletely understood; however, these genomic alterations could have implications for viral fitness, host adaptation, or virulence.

An analysis of the distinctions between the genomes of related ASFV strains exhibiting different virulence should provide insights into the critical factors that are important for virulence. A notable characteristic of the ASFV genome is its substantial abundance of multigene families. Especially, the MGF-360 and MGF-505/530 appear to play significant roles in viral tropism, virulence, and the suppressing type I interferon response. Nevertheless, there exists a substantial gap in understanding regarding the functional mechanisms of the encoded proteins that contribute to their proliferation and deletion. The ASFV strain OURT 88/3, which is known to have low virulence, is deficient in five MGF 360 and two MGF 505/530 members [7]. Also prior studies have suggested that the MGF 360-9L is associated with virulence through its antagonistic effects on the JAK/STAT signaling pathway, establishing it as a major virulence factor of ASFV [38]. The MGF-4L/5L, MGF 100-1R, and 285L do not appear to correlate directly with lower virulence, as these factors are also present and conserved in other highly virulent strains. Conversely, notable differences are observed in MGF 110-2L, -9L, and 86R which may contribute to lower virulence. A study involving isolates devoid of all MGF 110 families suggested that these factors may not be requisite for replication or have a role in virulence [39]. Further genomic factors related to virulence have to be considered.

Additionally, previous research has indicated recombination events in ASFV, including homologous recombination leading to genomic Indels, which contribute to viral genetic diversity, and recombination among different genotypes facilitated by recombination hotspots, resulting in diverse genetic strains [25, 40, 41]. Therefore, global cooperation for whole-genome sequencing of ASFVs from domestic pigs and wild boars is crucial to improving our understanding of ASFV epidemiology and transmission dynamics. In some regions of China and Vietnam, genotype I and II recombinant ASFV strains, characterized by high virulence, have emerged [42–44]. A theoretical basis for the duplex fluorescent PCR method to discriminate between genotypes I, II, and I and II recombinant strains has been provided [45]. Furthermore, a novel ASFV with a large-fragment deletion in the genome was detected in China, which has significantly prolonged onset and survival times and lower virulence [46]. In response to this challenge, several commercial attenuated vaccines directed at ASFV have recently been introduced and tested through on-farm trials in Vietnam, indicating a transition towards more comprehensive control measures.

Despite the large genetic deletion, both viruses isolated in this study did not show significant pathological or clinical differences in virulence compared to the first Korean ASFV Paju1/2019 strain [28], implying that the MGF 360-6L and MGF 360-21R genes may not be involved in swine virulence. Further research is necessary to investigate the mechanisms behind such large deletions and gene reorganization under natural conditions and their implications for changes in biological characteristics, particularly in protein functions.

Clinical signs commonly observed included fever, loss of appetite, depression, recumbency, and death, with additional symptoms like skin erythema, labored breathing, ocular discharge, diarrhea, and bloody diarrhea in some cases. These signs are not specific to ASF; however, the consistent occurrence of death among infected pigs, even in cases without clear clinical signs, was a significant outcome. Pathological examinations revealed specific lesions, such as markedly enlarged and hemorrhagic spleen infarction and gastrohepatic lymph nodes, consistently observed in infected pigs. However, the variability in pathological lesions among animals infected with highly virulent ASFV isolates emphasize the importance of considering all disease aspects in pathological examinations and conducting laboratory diagnoses even in the absence of clear ASF lesions. Mortality rates, clinical manifestations, survival periods, and necropsy findings were similar to those observed in other highly virulent ASFV strains.

In Group 1, pig 5 appeared to be infected with ASFV, but had low viral load in tissues, and symptoms of diarrhea, with histopathological findings suggesting possible salmonellosis. Despite these observations, the pig succumbed similarly to other pigs in the group. Notably, it showed joint edema, which is not characteristic of the acute form. We did not use SPF pigs for the experiment; instead, we purchased them from a commercial pig farm, where we suspected that they might have already been exposed to common bacterial infections due to the environmental conditions of the pig farm. Therefore, we believe that this pig was also infected with the acute ASF virus. Moreover, we did not detect the viral genome in oral swabs from pig 5 in Group 1 and there was no viremia in the samples. Swab sampling generally presented a better option for early detection than blood sampling. However, in this study, viral shedding through swab samples was less efficient than through blood samples due to the number of viral copies and early detection.

Based on the clinical course and virulence criteria for ASFV in pigs, two Korean ASFV isolates were identified as highly virulent, leading to an acute form of ASF. In this study, there were no significant changes in pathogenicity compared to Korean ASFV strains isolated from pig farms between 2019 and January 2023 [28, 33].

Additionally, in our recent study, we evaluated virulence based on different inoculation routes. For the direct contact route using cohabiting animals, pathogenicity appeared later than for other routes of inoculation, such as intramuscular, oral, and nasal; however, it still exhibited acute forms and remained highly contagious, which did not significantly affect virulence compared to other inoculation routes [47]. The study also confirmed that intramuscular inoculation was more favorable for early detection in virulence assessment and vaccine efficacy trials compared to oral and nasal inoculations. At the same time, however, it can be considered to conduct experiments with cohabiting animals to evaluate the transmissibility.

Recent research has examined the pathological lesions associated with experimental studies involving ASFV-infected pigs with viruses isolated from domestic pig farms where ASF outbreaks have occurred in South Korea; however, this study is the first to evaluate both viremia and pathomorphological lesions in the thoracic vertebrae of experimentally infected pigs exposed to Korean isolates.

While these isolates were derived from domestic pigs, it is important to consider that outbreaks on domestic farms may have resulted from spillover infections of ASFV strains present in wild boars. This implies that the ASFV strains circulating in South Korea are highly virulent and likely to persist, supporting the effectiveness of current control measures. Furthermore, the study has highlighted specific characteristics that could enhance the current surveillance system, such as targeted monitoring of deceased pigs, training programs for veterinary personnel conducting necropsies, and the selection of optimal sample types. In addition to the ASFV strains already circulating in South Korea, it is important to recognize that new ASFV variants introduced from other regions pose a serious threat. This challenge is compounded by the constantly changing ASF situation in neighboring countries.

## 5. Conclusions

The potential introduction of recombinant or low-virulence ASFV strains to domestic pig farms could lead to substantial alterations in the viral strains present in South Korea. Therefore, continuous efforts to elucidate the virulence of ASFV strains are essential. These endeavors are necessary to promptly detect any variations through international cooperation and support the development of effective control measures to combat the spread of ASFV in South Korea.

## Figures and Tables

**Table 1:** Summary of the virus information and genetic characterization results of the two representative ASFV isolates from outbreak pig farms in South Korea during 2023.

Sequence of outbreak	Date of outbreak	Strain nomination	Location (Province)	Herd size (Production type)	Classification	Sample type	Clinical signs	qPCR (Ct value)	HAD	p72 genotype	p54 genotype	CD2v serogroup	IGR I73R-I329L	CVR
33th	19 March 2023	ASFV/Korea/Pig/ Pocheon2/2023	Pocheon (Gyeonggi)	12,842 (fallow-to-finish)	Farmer's notification	Spleen	Abortion and Fetal death, death on sows	18.11	Positive	II	II	8	II	CVR1

37th	18 July 2023	ASFV/Korea/Pig/ Cheorwon2/2023	Cheorwon (Gangwon)	6,077 (fallow-to-finish)	Active surveillance	Blood	Anorexia on sows	23.09	Positive	II	II	8	II	CVR1

**Table 2:** Summary of genetic variations detected between two Korean African swine fever virus sequences from the reference strain, Georgia 2007/1 (NC_044959), except large size deletion.

Gene	Nucleotide substitution^a^ (Amino acid change)	ASFV/Korea/Pig/ Pocheon2/2023	ASFV/Korea/Pig/ Cheorwon2/2023
MGF 110-1L	C7059T (W197*⁣*^*∗*^)	T	T
MGF 360-10L	T26425C (N329S)	C	C
MGF 360-10L	A27046G (I122T)	A	G
MGF 360-10L	C27183G (Syn^c^)	G	G
MGF 505-9R	A44576G (K323E)	G	G
A179L, A137R IGR^b^	A55405G (noncoding)	G	G
C129R	G82005A (Syn)	G	A
C84L	T82565C (Q5R)	C	T
C475L	T87044C (I360V)	C	C
B602L, B385R IGR	103315G insertion (noncoding)	G insertion	G insertion
B407L	G107354A (Syn)	A	G
B407L	G107992A (H170Y)	G	A
CP2475L	A118825G (Syn)	G	A
NP419L	T134514C (N414S)	C	C
D1133L	C144022T (E239K)	T	T
D1133L	C144396T (R114H)	C	T
I267L	T170862A (I195F)	A	A
I73R, I329L IGR	GAATATATAG insertion (noncoding)	GAATATATAG insertion	GAATATATAG insertion

^a^Homopolymeric regions and inverted terminal repeats were excluded from the analysis due to their high error rates in sequencing.

^b^Intergenic region.

^c^Synonymous mutation.

*⁣* ^*∗*^Stop codon.

**Table 3:** Comparison of clinical signs between two ASFV isolates infected groups from domestic pig farms during 2023.

Clinical signs	*n* Animals Affected		Overall
	Group 1	Group 2	
Died or euthanized (dpi)	7.4 ± 0.5	9.4 ± 0.5	—
Fever (>40°C)	5/5	5/5	10/10 (100%)
Loss appetite/anorexia	5/5	5/5	10/10 (100%)
Depression/recumbency	5/5	5/5	10/10 (100%)
Skin hemorrhage	0/5	2/5	2/10 (20%)
Joint swelling	1/5	0/5	1/10 (10%)
Wheezing/coughing	0/5	1/5	1/10 (10%)
Ocular discharge	2/5	5/5	7/10 (70%)
Diarrhea	2/5	0/5	2/10 (20%)
Hematuria/epistaxia	0/5	1/5	1/10 (10%)
Vomit	0/5	0/5	0/10 (0%)

**Table 4:** Comparison of clinical characteristics, viremia, and viral load in the shedding between groups of infected pigs.

Group (number of infected pigs)	ASFV strain	Total days survival (±SD)	Days to onset of pyrexia (±SD)	Clinical signs		Viremia		Viral load in the shedding					
								Oral		Nasal		Rectal	
				Days to the onset (±SD)	Maximum score*⁣*^*∗*^ (±SD)	Days to the onset (±SD)	Maximum titer*⁣*^*∗*^ (±SD)	Days to the onset (±SD)	Maximum titer*⁣*^*∗*^ (±SD)	Days to the onset (±SD)	Maximum titer*⁣*^*∗*^ (±SD)	Days to the onset (±SD)	Maximum titer*⁣*^*∗*^ (±SD)
1 (*n* = 5)	Korea/Pig/ Pocheon2/ 2023	7.4 (±0.5)	4.2 (±1.8)	6.4 (±0.5)	12.2 (±1.1)	3.4 (±0.9)	6.6 (±0.8)	4.8 (±0.5)	2.3 (±1.3)	5.0 (±0.7)	3.0 (±1.1)	5.0 (±0.7)	3.4 (±0.6)

2 (*n* = 5)	Korea/Pig/ Cheorwon2/ 2023	9.4 (±0.5)	4.4 (±0.9)	6.6 (±1.1)	14.6 (±1.7)	3.0 (±0.0)	7.0 (±0.2)	4.2 (±0.4)	4.6 (±0.5)	4.8 (±0.4)	5.2 (±0.1)	4.8 (±0.4)	4.8 (±0.2)

*Note:* Five pigs per groups were inoculated intramuscularly with same volume of viruses which two ASFV isolates from domestic pig farms during 2023.

Abbreviation: SD, standard deviation.

*⁣* ^*∗*^log_10_ genome copies/µL.

**Table 5:** Comparison of the gross lesions between two ASFV isolates infected groups from domestic pig farms during 2023 by macroscopic findings.

Gross pathological criteria					*n* Animals affected		Overall
					Group 1	Group 2	
	Tissues			Macroscopic findings	Annotations		
Gross lesions	Lymphoid system	Tonsil		Erythema	2/5	2/5	4/10 (40%)
		Spleen		Enlargement	4/5	5/5	9/10 (90%)
				Hemorrhagic infarction	5/5	5/5	10/10 (100%)
		Lymph nodes	Submandibular Tracheobronchial Gastric Gastrohepatic Renal Mesenteric	Erythema	4/5	5/5	9/10 (90%)
				Enlargement	5/5	5/5	10/10 (100%)
				Hemorrhagic infarction	3/5	5/5	8/10 (80%)
	Cardiorespiratory system	Heart		Epi-/Endocardial hemorrhage and petechiae	1/5	1/5	2/10 (20%)
		Lungs		Erythema	4/5	4/5	8/10 (80%)
				Interstitial pneumonia	1/5	2/5	3/10 (30%)
				Interlobular edema	2/5	2/5	4/10 (40%)
	Alimentary system	Liver		Petechiae	1/5	2/5	3/10 (30%)
		Intestine		Petechiae in serosa	0/5	2/5	2/10 (20%)
	Urinary system	Kidney		Hemorrhagic renal medulla	1/5	3/5	4/10 (40%)
				Petechiae on renal cortex	1/5	5/5	6/10 (60%)
		Urinary bladder		Petechiae in mucosa	0/5	4/5	4/10 (40%)

**Table 6:** Comparison of histopathological analysis in dead pigs from infected two isolates.

Tissues			Microscopic findings	Group 1					Group 2					Total frequency
				1	2	3	4	5	1	2	3	4	5	
Lymphoid system	Tonsil		Necrosis of lymphocytes with hemorrhages	++	++	−	+++	−	++	++	−	−	++	6/10 (60%)
	Spleen		Necrotizing splenitis with hemorrhages	+++	+++	+++	+++	+	+++	++	+++	++	+++	10/10 (100%)
	Lymph nodes	Submandibular	Necrotizing lymphadenitis	+++	+++	+++	+++	−	+++	+++	+++	+++	+++	9/10 (100%)
		Tracheobronchial	Necrotizing lymphadenitis with hemorrhages	+++	++	+++	−	−	+++	+++	++	+++	+++	8/10 (80%)
		Gastric	Necrotizing lymphadenitis with hemorrhages	+++	+++	+++	++	−	+++	++	+++	+++	+++	9/10 (90%)
		Gastrohepatic	Necrotizing lymphadenitis with hemorrhages	+++	+++	+++	+++	−	+++	+++	+++	+++	+++	9/10 (90%)
		Renal	Necrotizing lymphadenitis with hemorrhages	+++	+++	+++	+	−	++	++	+++	+++	+++	9/10 (90%)
		Mesenteric	Necrotizing lymphadenitis	+++	++	+++	+++	−	+++	++	++	+++	+++	9/10 (90%)
	Peyer's patch		Necrotizing lymphadenitis	−	−	−	++	−	−	−	−	−	−	1/10 (10%)
Nervous system	Thoracic vertebrae		Blood vessel degeneration and necrosis with perivascular inflammatory cells infiltration	++	++	+	++	−	+	+	++	+	+	9/10 (90%)
Cardiorespiratory system	Heart		Myocardial hemorrhage with necrosis of blood vessel in epicardium	+++	−	−	−	−	−	−	−	−	−	1/10 (10%)
	Lungs		Interstitial pneumonia with edema	+	+	+	++	−	+	++	++	++	+	9/10 (90%)
Alimentary system	Liver		Hepatic cell necrosis	+	−	+	+	−	++	++	++	+	+	8/10 (80%)
Urinary system	Kidneys		Hemorrhage in renal pelvis and renal cortex congestion	++	+	+	+	−	++	++	++	++	++	9/10 (90%)
	Urinary bladder		Submucosal hemorrhages	−	−	−	−	−	−	+	−	−	−	1/10 (10%)

*Note:* Gross lesion scores were calculated according to previously published guidelines (no lesion (−), mild (+), moderate (++), and severe (+++)).

## Data Availability

The dataset generated in this study is available from the first author and corresponding authors on reasonable request.

## Supporting Information

Supporting Information See Table S1–S3 and Figure S1 in the Supporting Information for comprehensive analysis. Table S1: Primers for Sanger sequencing of partial MGF 360-21R and MGF 360-1 La. Table S2: List of reference African swine fever virus sequences used in phylogenetic analysis. Table S3: Next-generation sequencing results of two ASFV isolates analyzed in this study. Figure S1: Comparison of tissue viral load in dead pigs from infected two isolates.
