# Supplementary material for: Pathological Characterization of African Swine Fever Viruses With Genetic Deletions Detected in South Korea
Source: Transbound Emerg Dis. 2025 May 6;2025:9917280. doi: 10.1155/tbed/9917280 (PMC12074837; doi:10.1155/tbed/9917280)
Supplement: Supporting Information — See Table S1–S3 and Figure S1 in the Supporting Information for comprehensive analysis. Table S1: Primers for Sanger sequencing of partial MGF 360-21R and MGF 360-1 La. Table S2: List of reference African swine fever virus sequences used in phylogenetic analysis. Table S3: Next-generation sequencing results of two ASFV isolates analyzed in this study. Figure S1: Comparison of tissue viral load in dead pigs from infected two isolates. [file 9917280.f1.docx]

Supporting materials

S1 Table. Primers for sanger sequencing of partial MGF 360-21R and MGF 360-1 La

| **Target** | **Primer** | **Sequence** | **Length** |
| --- | --- | --- | --- |
| MGF 360-21R(deletion) and MGF 360-1 La(replace) | CWDel_Re_F | 5’-GTTTATAGGGGCTTGAAAAAC-3’ | 1,180bp |
|  | CWDel_Re_R | 5’-TTTCCTGTTAGGTGGTTTG-3’ |  |

S2 Table. List of reference African swine fever virus sequences used in phylogenetic analysis

| Strain name | Length | Country | Collection Date | Genebank Accession number |
| --- | --- | --- | --- | --- |
| ASFV_Georgia_2007/1 | 190584 | Georgia | 2007 | NC_044959 |
| ASFV_Georgia_2007/1 | 190584 | Georgia | 2007 | FR682468 |
| Georgia_2008/1 | 189465 | Georgia | 2018 | MH910495 |
| ASFV/Kabardino-Balkaria_19/WB-964 | 189252 | Russia | 2019-03-26 | MT459800 |
| Arm/07/CBM/c2 | 190145 | Armenia | 2017 | LR812933 |
| Kashino_04/13 | 189387 | Russia | 2013-03-01 | KJ747406 |
| ASFV/LT14/1490 | 189399 | Lithuania | 2014-01-01 | MK628478 |
| ASFV/POL/2015/Podlaskie | 189404 | Poland | 2015 | MH681419 |
| ASFV_Moldova_2017/1 | 190598 | Moldova | 2017 | LR722599 |
| ASFV_CzechRepublic_2017/1 | 190594 | Czech Republic | 2017 | LR722600 |
| Belgium/Etalle/wb/2018 | 190202 | Belgium | 2018-09-10 | MK543947 |
| 20355/RM/2022_Italy | 190590 | Italy | 2022 | OP605386 |
| 21730_1474/RM/2022 | 190590 | Italy | 2022 | OR460730 |
| 7540/22 | 190120 | Serbia | 2022 | OR660699 |
| DG_6511_21_ | 190117 | Serbia | 2021 | OR660695 |
| DG_6314_19 | 190108 | Serbia | 2019 | OR660696 |
| 47169.16_1499/GE/2022_Ita | 190586 | Italy | 2022 | OR460732 |
| 50665.8_2167/AL/2022 | 190599 | Italy | 2022 | OR460739 |
| 2802/AL/2022_Italy | 190596 | Italy | 2022 | ON108571 |
| 47169.12_1495/GE/2022 | 190599 | Italy | 2022 | OR460731 |
| DG_6759_19 | 190047 | Serbia | 2019 | OR660698 |
| ASFV_Belgium_2018/1 | 190599 | Belgium | 2017 | LR536725 |
| ASFV/Belgorodskaya_2021/DP-11838 | 189379 | Russia | 2021 | PP982230 |
| ASFV/Sverdlovskaya_2021/DP-9914 | 189394 | Russia | 2021 | PP982245 |
| ASFV/Bryanskaya_2021/DP-8823 | 189228 | Russia | 2021 | PP982233 |
| ASFV/Amur_2022/WB-909 | 188599 | Russia | 2022 | PP982228 |
| ASFV/Belgorodskaya_2021/DP-11869 | 189225 | Russia | 2021 | PP982231 |
| Pol17_04461_C210 | 189401 | Poland | 2017-12-01 | MG939588 |
| Pol17_05838_C220 | 189393 | Poland | 2017-12-01 | MG939589 |
| Pol16_20186_o7 | 189401 | Poland | 2017-12-01 | MG939583 |
| Pol18_28298_O111 | 189409 | Poland | 2019 | MT847621 |
| ASFV/Kaliningrad_18/WB-12523 | 189111 | Russia | 2018-08-07 | OM966714 |
| ASFV/Kaliningrad_17/WB-13869 | 189129 | Russia | 2017-11-07 | OM799941 |
| ASFV/Kaliningrad_19/WB-10168 | 189131 | Russia | 2019-05-13 | OM966719 |
| ASFV/Kaliningrad_18/WB-9763 | 189125 | Russia | 2018-07-07 | OM966717 |
| ASFV/Khabarovsk_2021/WB-3967 | 190594 | Russia | 2021 | PP982237 |
| Pol17_31177_O81 | 189422 | Poland | 2019 | MT847622 |
| Pol17_55892_C754 | 189414 | Poland | 2019 | MT847620 |
| ASFV_Germany_2020/1 | 190592 | Germany | 2020 | LR899193 |
| 2021ASP02148 | 190595 | Germany | 2021-04-29 | OX376257 |
| 2020ASP02103 | 190595 | Germany | 2020-10-07 | OX376261 |
| 2021ASP02665 | 190611 | Germany | 2021-05-11 | OX376259 |
| 2021ASP00921 | 190597 | Germany | 2021-02-22 | OX376262 |
| 2021ASP03740 | 190592 | Germany | 2021-07-29 | OX376272 |
| 2021ASP00902 | 190595 | Germany | 2021-02-18 | OX376255 |
| 2021ASP01919 | 190595 | Germany | 2021-04-19 | OX376251 |
| Korea/Pig/Ganghwa5/2019 | 187848 | South Korea | 2019-09-26 | OR145822 |
| DB/LN/2018 | 189404 | China | 2018-09-01 | MK333181 |
| ASFV/Zabaykali/WB-5314/2020 | 189248 | Russia | 2020-08-04 | MZ325862 |
| ASF-MNG19 | 187868 | Mongolia | 2019 | OP467597 |
| China/2018/AnhuiXCGQ | 189393 | China | 2018-09-02 | MK128995 |
| ASFV2020-015-B | 190565 | Philippines | 2020-05-05 | MW791755 |
| ASFV/Timor-Leste/2019/1 | 192237 | Timor-Leste | 2019 | MW396979 |
| ASFV_JS | 192224 | China | 2022 | OR180113 |
| ASFV/Khabarovsk_2020/WB-11558 | 190586 | Russia | 2020 | PP982236 |
| ASFV/Khabarovsk_2022/WB-1650 | 190591 | Russia | 2022 | PP982239 |
| ASFV/Amur_2022/WB-905 | 188582 | Russia | 2022 | PP982227 |
| ASFV/Amur_2021/WB-10591 | 190567 | Russia | 2021 | PP982225 |
| ASFV2020-018-B | 190559 | Philippines | 2020-02-24 | MW791756 |
| ASFV/pig/China/CAS19-01/2019 | 189405 | China | 2019-01-02 | MN172368 |
| ASFV/Amur_19/WB-6905 | 189248 | Russia | 2019-08-29 | MW306190 |
| ASFV2020-013-B | 190571 | Philippines | 2020-03-24 | MW791753 |
| ASFV_serotype_8_genotype_2 | 189487 | Viet Nam | 2021 | ON402789 |
| Korea/Pig/Goseong/2021 | 187854 | South Korea | 2021-08-07 | OR145831 |
| Korea/Pig/Yeongwol/2021 | 187850 | South Korea | 2021-05-04 | OR145830 |
| Korea/Pig/Inje1/2021 | 187849 | South Korea | 2021-10-05 | OR145832 |
| Mon_Dom | 189389 | Mongolia | 2019-01-01 | OR271566 |
| ASFV2020-014-B | 190563 | Philippines | 2020-05-02 | MW791754 |
| CADC_HN09 | 190257 | China | 2019 | MZ614662 |
| PAN20211A | 189514 | Philippines | 2021 | PP737709 |
| A4 | 192265 | Philippines | 2021-08-01 | ON963982 |
| ASFV2019-003-B | 190569 | Philippines | 2019-12-06 | MW791760 |
| Korea/Pig/Gimpo2/2019 | 187848 | South Korea | 2019-10-02 | OR145826 |
| Korea/Pig/Gimpo1/2019 | 187848 | South Korea | 2019-09-23 | OR031244 |
| Korea/Pig/Ganghwa2/2019 | 187849 | South Korea | 2019-09-25 | OR145819 |
| ASFV2020-021-B | 190565 | Philippines | 2020-04-24 | MW791759 |
| Korea/Pig/Ganghwa4/2019 | 187848 | South Korea | 2019-09-26 | OR145821 |
| ASFV2020-003-B | 190565 | Philippines | 2020-01-29 | MW791761 |
| Korea/Pig/Ganghwa3/2019 | 187848 | South Korea | 2019-09-25 | OR145820 |
| ASFV/Primorsky_19/WB-6723 | 189256 | Russia | 2019-08-28 | MW306191 |
| China/LN/2018/1 | 189397 | China | 2018-08-03 | OP856591 |
| SY-1 | 189404 | China | 2020 | OM161110 |
| ASFV2020-020-B | 190561 | Philippines | 2020-06-18 | MW791758 |
| ASFV_Wuhan_2019-2 | 190576 | China | 2019-08-19 | MN393477 |
| ASFV_Wuhan_2019-1 | 190576 | China | 2019-08-19 | MN393476 |
| Pig/HLJ/2018 | 189404 | China | 2018-09-05 | MK333180 |
| Yangzhou | 187951 | China | 2021-10-01 | ON456300 |
| GZ201801_2 | 189401 | China | 2018 | ON263123 |
| ASFV/Amur_2022/WB-911 | 188592 | Russia | 2022 | PP982229 |
| ASFV/Khabarovsk_2020/DP-11562 | 190585 | Russia | 2020 | PP982235 |
| ASFV/Primorsky_2021/DP-9778 | 190588 | Russia | 2021 | PP982242 |
| Korea/Pig/Ganghwa1/2019 | 187848 | South Korea | 2019-09-23 | OR145818 |
| ASFV/JAO_2020/DP-6768 | 190583 | Russia | 2020 | PP982234 |
| IND/AS/SD-02/2020 | 190517 | India | 2020-04-01 | OL692743 |
| IND/AR/SD-61/2020 | 190572 | India | 2020-04-01 | OL692744 |
| ABTCVSCK_ASF001 | 190598 | India | 2020 | OM481275 |
| ABTCVSCK_ASF007 | 190595 | India | 2021 | OM481276 |
| China/LN/2018/2 | 189394 | China | 2018-10-16 | OR958825 |
| ASFV_HU_2018 | 190601 | Hungary | 2018-04-24 | MN715134 |
| ASFV-wbShX01 | 189401 | China | 2019-11-01 | MW033528 |
| Korea/pig/PaJu1/2019 | 187848 | South Korea | 2019-09-16 | MT748042 |
| MSR2022S1 | 189514 | Philippines | 2022 | PP737711 |
| ASFV2020-008-B | 190565 | Philippines | 2020-02-27 | MW791752 |
| Korea/HC224/2020 | 188645 | South Korea | 2020 | OP628183 |
| Korea/CW714/2020 | 188784 | South Korea | 2020 | OR162436 |
| Korea/YC1/2019 | 188950 | South Korea | 2019-10-02 | ON075797 |
| Korea/Pig/Yeuncheon2/2019 | 187848 | South Korea | 2019-10-09 | OR145827 |
| S-S-VR-413000-00008 | 189429 | South Korea | 2020 | OR159218 |
| Korea/Pig/Hongcheon/2021 | 187848 | South Korea | 2021-08-25 | OR145833 |
| Korea/pig/Yeoncheon1/2019 | 187848 | South Korea | 2019-09-17 | MW049116 |
| Korea/Pig/Paju4/2019 | 187849 | South Korea | 2019-10-01 | OR145824 |
| Korea/Pig/Paju3/2019 | 187849 | South Korea | 2019-10-01 | OR145823 |
| Korea/Pig/Paju2/2019 | 187848 | South Korea | 2019-10-02 | OR145817 |
| Korea/Pig/Paju5/2019 | 187848 | South Korea | 2019-10-02 | OR145825 |
| Korea/Pig/Hwacheon2/2020 | 187848 | South Korea | 2020-10-09 | OR145829 |
| Korea/Pig/Hwacheon1/2020 | 187847 | South Korea | 2020-10-08 | OR145828 |
| Korea/PC1432/2021 | 188585 | South Korea | 2021 | OR180305 |
| S-S-VR-413000-00002 | 189400 | South Korea | 2020 | OR159219 |
| S-S-VR-413000-00015 | 189411 | South Korea | 2020 | OR159217 |

S3 Table. Next-generation sequencing results of two ASFV isolates analyzed in this study

| Strain | Total number of reads | Percent of reads mapped to ASFV genome^A^ | Mean depth of coverage^B^ | Recovered genome size | GenBank acc. Number |
| --- | --- | --- | --- | --- | --- |
| ASFV/Korea/Pig/Pocheon2/2023 | 2,981,620 | 2,319,361 | 1455 | 185,917 | PQ185530 |
| ASFV/Korea/Pig/Cheorwon2/2023 | 2,554,652 | 1,857,923 | 1033.4 | 188,148 | PQ185531 |

The number of total reads and percent of reads mapped to ASFV genome. ^A^Taxonomic classification of sequencing reads whereas assigned using KRAKEN2 program to calculate the percentage of ASFV genomes to the total reads. ^B^The mean number of reads per each nucleotide site.

S1 Figure. Comparison of tissue viral load in dead pigs from infected two isolates. The red triangle represents the Pocheon isolate and blue rectangle represents the Cheorwon isolate and mean (solid lines) viral copy number per microliter in two groups are shown. LN, lymph node.
